# Supplementary material for: Utilizing computer vision for facial behavior analysis in schizophrenia studies: A systematic review
Source: PLoS One. 2022 Apr 8;17(4):e0266828. doi: 10.1371/journal.pone.0266828 (PMC8992987; doi:10.1371/journal.pone.0266828)
Supplement: S1 Table — (PDF) [file pone.0266828.s001.pdf]

Supplementary Table 1. Data processing steps.

Data processing steps.

Article	Data processing steps.
[32]	Each image was classified using four SVMs for four facial expressions respectively, with wavelet decomposition features from RVD map, trained on auxiliary actor facial expression dataset of 181 images [69].
[33,34]	Faces were detected with an Adaboost based detector [70]; Fiducial landmarks identified using Active Appearance Model [71]; 3D facial regions created from the 2D shape; Validity of the 2D and 3D features were evaluated by using them to classify each expression into five emotions using LDA and KNN, trained on the actor dataset [69].
[35]	Faces and landmarks were detected and 2D geometric features were calculated similarly to [33]. Five SVMs were trained on the actor dataset [69].
[38]	ROIs (head and body) were manually predefined and located.
[36,37]	Face detected with Viola-Jones face detector [70], and facial landmark identified with Active Shape Model [72]; Geometric features and Gabor wavelet response in ROIs as texture features were used as input for 15 Adaboost [73] classifiers, trained with 3419 face images labeled with FACS ratings, to detect 15 AUs independently.
[39]	3D wire-frame representation of participants' movements using Vicon iQ software; Identification of the listener and speaker based on head orientation; identification of nodding and gesture based on thresholding head and hand movement speed.
[45]	23 AUs were recognized using FaceShift software.
[47]	23 AUs were recognized using FaceShift software; Centroids of the clusters generated by k-means was used as the prototype expressions.
[46]	23 AUs were recognized using FaceShift software; Five video-level facial activity features were calculated described in [45].
[44]	20 AUs were recognized using OpenFace [21].
[43]	Markers were placed on the face of the participant.
[41,42]	Body was detected with SSD [74] and face was then detected with SmileNet [75]; Ten AUs were identified using ten VGG16 networks trained on EmotioNet [23], ExpW [76], CelebA [77] and CEW dataset [78]; Smile was recognized using SmileNet [75].
[40]	Seven emotions were recognized with SVM and histogram of frequencies features described in [79].
[48]	Location of the faces were recognized with OpenFace [21].
